# Decreased TOB1 expression and increased phosphorylation of nuclear TOB1 promotes gastric cancer

**DOI:** 10.18632/oncotarget.20749

**Published:** 2017-09-08

**Authors:** Rongwei Guan, Lei Peng, Dong Wang, Hongjie He, Dexu Wang, Rui Zhang, Hui Wang, Huiting Hao, Jian Zhang, He Song, Shuning Sui, Xiangning Meng, Xiaobo Cui, Jing Bai, Wenjing Sun, Songbin Fu, Jingcui Yu

**Affiliations:** ^1^ Scientific Research Centre, The Second Affiliated Hospital of Harbin Medical University, Harbin 150081, China; ^2^ Laboratory of Medical Genetics, Harbin Medical University, Harbin 150081, China

**Keywords:** gastric cancer, TOB-1, p-TOB1, subcellular localization, anti-proliferative activity

## Abstract

TOB1, a member of the BTG/TOB protein family, inhibits tumor cell proliferation. We previously observed down-regulation and phosphorylation of TOB1 in gastric cancer (GC). Here, we examined the subcellular distribution and clinical significance of TOB1 expression and phosphorylation in GC. Immunohistochemical analysis of 341 primary GC and corresponding normal gastric tissue samples demonstrated that nuclear TOB1 expression was lower in GC than normal tissue (80.4% vs. 92.4%), and decreased nuclear TOB1 expression correlated with high TNM stage. By contrast, phosphorylation of nuclear TOB1 was higher in GC than normal gastric tissue (66.0% vs. 36.4%), and was associated with poorly differentiated and high TNM stage tumors. Patients with intestinal type GC and increased nuclear TOB1 phosphorylation had poor overall survival. Multivariate survival analysis indicated the nuclear concentration of phosphorylated TOB1 was an independent prognostic factor for intestinal type GC. Overexpression of TOB1 containing mutations in its nuclear export signal inhibited GC cell proliferation, migration, and invasion compared to cells expressing TOB1 with the nuclear localization signal. Thus, decreased TOB1 expression and increased phosphorylation of nuclear TOB1 is associated with aggressive tumor behavior and poor prognosis in intestinal type GC. Additionally, TOB1 nuclear retention is critical for its anti-proliferative activity.

## INTRODUCTION

Human transducer of ERBB2, 1 (TOB1) is a member of the TOB/BTG family of anti-proliferative proteins [1]. It binds to transcription factors in the nucleus and regulates gene expression. Reduced TOB1 expression and altered phosphorylation has been observed in various cancers including lung [2], thyroid [3], breast [4], pancreatic [5], and squamous cell carcinoma of the skin [6]. Loss of TOB1 expression promoted lung cancer cell proliferation, invasion, and metastasis [7]. Previous data suggests TOB1 is a tumor suppressor that inhibits cancer cell proliferation through various signaling pathways [8].

We recently identified several allelic deletions on chromosomes 17 and 18 in 45 patients with primary gastric cancer (GC) using loss of heterozygosity analysis and microsatellite markers. *TOB1* lies in one of these regions (17q21.3-22) on the long arm of chromosome 17 [9, 10]. We analyzed TOB1 expression and phosphorylation in four GC cell lines and tissue specimens from 97 patients with primary GC. Down-regulation of TOB1 expression and accumulation of phosphorylated TOB1 (p-TOB1) promoted carcinogenesis. Thus, inactivation of TOB1 may play a critical role in GC [11]. Overexpression of TOB1 inhibited GC progression by activating Smad4 and inhibiting β-catenin-mediated signaling [12]. Li et al. demonstrated that TOB1 was a target of miR-25, which repressed TOB1 expression by targeting the TOB1 3′-UTR in GC [13]. A single nucleotide polymorphism in miR-25, rs41274221, disrupted miR-25 regulation of TOB1 and was protective against GC [14].

The anti-proliferative effects of TOB1 may be associated with its subcellular distribution. Kawamura-Tsuzuku et al. showed that nuclear localization of TOB1 was important for its anti-proliferative effects in NIH3T3 cells [15]. However, Maekawa et al. suggested that the anti-proliferative effects were mediated by cytoplasmic TOB1 in these cells [16]. Reduced TOB1 expression in the cytoplasm was associated with the clinicopathological characteristics of 90 GC patients [17].

We investigated the relationship between the subcellular distribution of TOB1/p-TOB1 and the clinical prognosis of GC patients. Additionally, we investigated the role of the subcellular localization of TOB1/p-TOB1 in gastric carcinogensis. Our results indicate that decreased TOB1 expression and increased nuclear p-TOB1 were associated with a malignant tumor phenotype and poor survival in GC patients. Nuclear localization of TOB1 is critical for its anti-proliferative effects in GC cells.

## RESULTS

### Decreased expression and increased phosphorylation of TOB1 in the nuclei of primary GC cells

**Figure 1:**
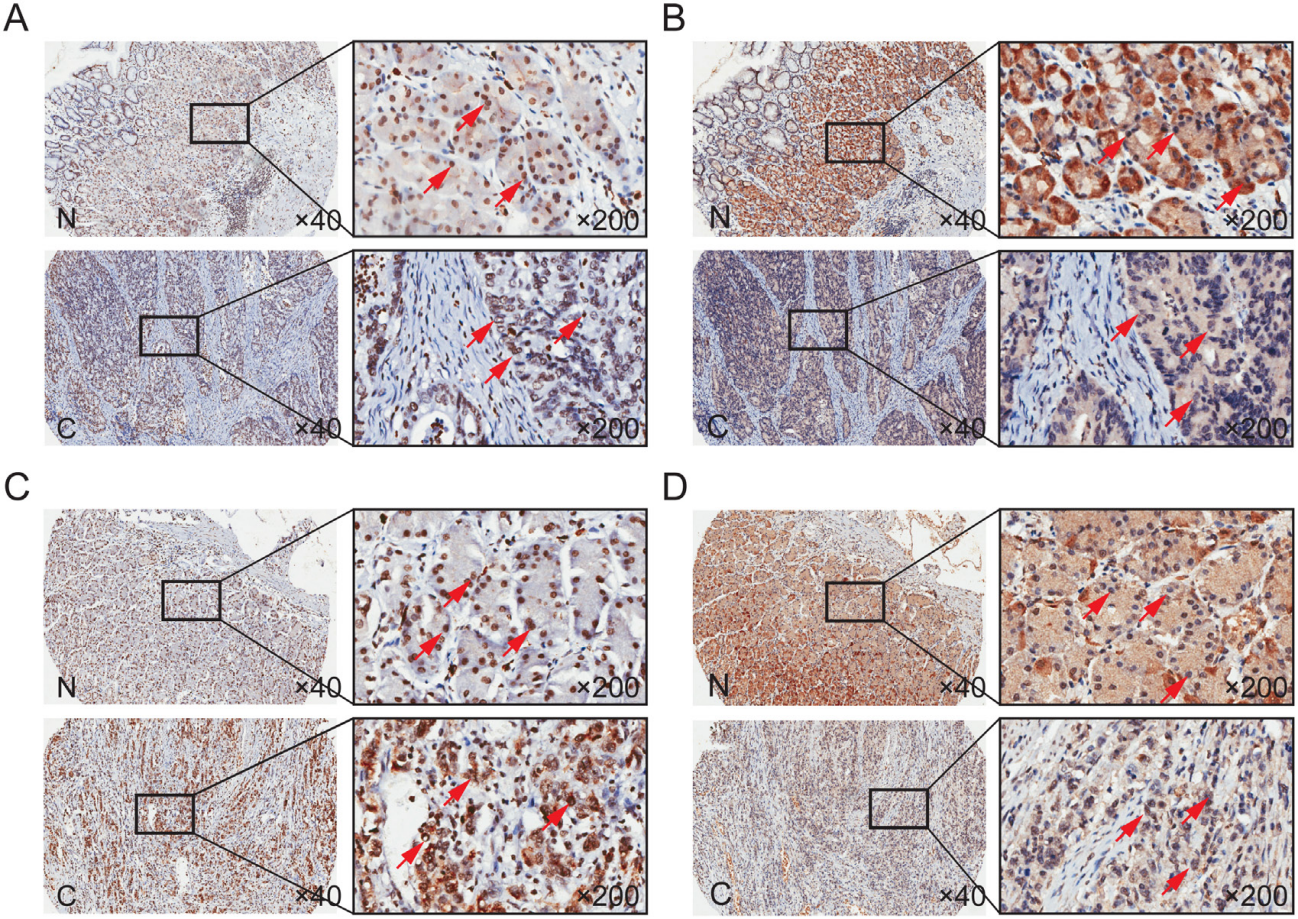
TOB1 and p-TOB1 protein expression in GC and NG tissue **(A** and **C)** Total TOB1 protein. **(B** and **D)** p-TOB1 protein. (A and B) Intestinal type. (C and D) Diffuse type. N, noncancerous gastric tissue; C, GC tissue.

We examined TOB1 and p-TOB1 levels in the nuclei and cytoplasm of GC cells in tissue microarrays (TMAs) that included 341 primary GC patients. Representative images of the immunohistochemical staining of TOB1 and p-TOB1 in cancerous and noncancerous gastric tissue (NG) obtained from intestinal or diffuse type GC patients are shown in Figure 1. TOB1 and p-TOB1 levels in GC tissue were compared with those in corresponding NG tissue. TOB1 was primarily detected in the nuclei of GC and NG cells (Figure 1A and 1C). The expression of nuclear TOB1 was lower in GCs (80.4%, 274/341) compared to NG tissues (92.4%, 315/341) (*P* = 0.000) (Table 1). We primarily detected p-TOB1 in the nuclei of GC cells and in the cytoplasm of normal gastric mucosa cells (Figure 1B and 1D, and Table 1). The expression of p-TOB1 was higher in the nuclei of GC (66.0%, 225/341) compared to NG cells (36.4%, 124/341) (*P* = 0.000), suggesting that p-TOB1 accumulates in the nuclei of GC cells.

**Table 1:** Subcellular distribution of TOB1 and p-TOB1 in GC cells

	Subcellular distribution	GC (n=341)	NG (n=341)	*P* value
TOB1	Cytoplasm	67 (19.6%)	26 (7.6%)	0.000***
	Nucleus	274 (80.4%)	315 (92.4%)	
p-TOB1	Cytoplasm	116 (34.0%)	217 (63.6%)	0.000***
	Nucleus	225 (66.0%)	124 (36.4%)	

### The association between decreased TOB1 expression and increased p-TOB1 nuclear accumulation and the clinical characteristics of GC patients

We next analyzed the association between TOB1 and p-TOB1 levels (Table 2) and the clinicopathological characteristics of 341 GC patients (Supplementary Table 1). Nuclear TOB1 expression was reduced in GC patients with lymph node metastasis (N0 vs. ≥ N1, *P* = 0.008), distant metastasis (M0 vs. M1, *P* = 0.007), and high TNM stage (I and II vs. III and IV, *P* = 0.043). In contrast, the expression of p-TOB1 in GC cell nuclei was associated with TNM stage, depth of invasion, and differentiation of the tumor. Higher levels of nuclear p-TOB1 were observed in GC patients with poorly differentiated tumors (G3 and G4, *P* = 0.027), deep tumor invasion (T3 and T4, *P* = 0.013), or high TNM stage (III and IV, *P* = 0.043) compared to GC patients with moderately or well differentiated tumors (G1 and G2), superficial tumor invasion (T1 and T2), or low TNM stage (I and II).

**Table 2:** Association between TOB1 and p-TOB1 subcellular distribution and the clinicopathological characteristics of GC patients

	TOB1 distribution		*P* value	p-TOB1 distribution		*P* value
	Cytoplasm	Nucleus		Cytoplasm	Nucleus	
**Age**			0.202			0.275
≤65 (n=196)	34(17.3%)	162(82.7%)		71(36.2%)	125(63.8%)	
>65 (n=144)	33(22.9%)	111(77.1%)		44(30.6%)	100(69.4%)	
**Gender**			0.244			0.570
Male (n=226)	40(17.7%)	186(82.3%)		79(35.0%)	147(65.0%)	
Female (n=113)	26(23.0%)	87(77.0%)		36(31.9%)	77(68.1%)	
**Tumor size (cm)**			0.303			0.857
≤5 (n=177)	31(17.5%)	146(82.5%)		61(34.5%)	116(65.5%)	
>5 (n=164)	36(22.0%)	128(78.0%)		55(33.5%)	109(66.5%)	
**Grade**			0.305			0.027*
G1-G2 (n=154)	34(22.1%)	120(77.9%)		62(40.3%)	92(59.7%)	
G3-G4 (n=187)	33(17.6%)	154(82.4%)		54(28.9%)	133(71.1%)	
**Lauren’s classification**			0.756			0.072
Intestinal (n=195)	40(20.5%)	155(79.5%)		76(39.0%)	119(61.0%)	
Diffuse (n=94)	16(17.0%)	78(83.0%)		23(24.5%)	71(75.5%)	
Mixed (n=49)	11(22.4%)	38(77.6%)		16(32.7%)	33(67.3%)	
Else (n=1)	0(0.0%)	1(100.0%)		0(0.0%)	1(100.0%)	
**T stage**			0.915			0.013*
T1-T2 (n=58)	11(19.0%)	47(81.0%)		28(48.3%)	30(51.7%)	
T3-T4 (n=281)	55(19.6%)	226(80.4%)		88(31.3%)	193(68.7%)	
**N stage**			0.008**			0.494
N0 (n=95)	10(10.5%)	85(89.5%)		35(36.8%)	60(63.2%)	
≥N1 (n=246)	57(23.2%)	189(76.8%)		81(32.9%)	165(67.1%)	
**M stage**			0.007**			0.241
M0 (n=326)	60(18.4%)	266(81.6%)		113(34.7%)	213(65.3%)	
M1 (n=15)	7(46.7%)	8(53.3%)		3(20.0%)	12(80.0%)	
**TNM stage**			0.043*			0.043*
I-II (n=139)	20(14.4%)	119(85.6%)		56(40.3%)	83(59.7%)	
III-IV (n=202)	47(23.3%)	155(76.7%)		60(34.0%)	142(66.0%)	

### The association between the nuclear concentration of p-TOB1 and the prognosis of intestinal type GC patients

**Figure 2:**
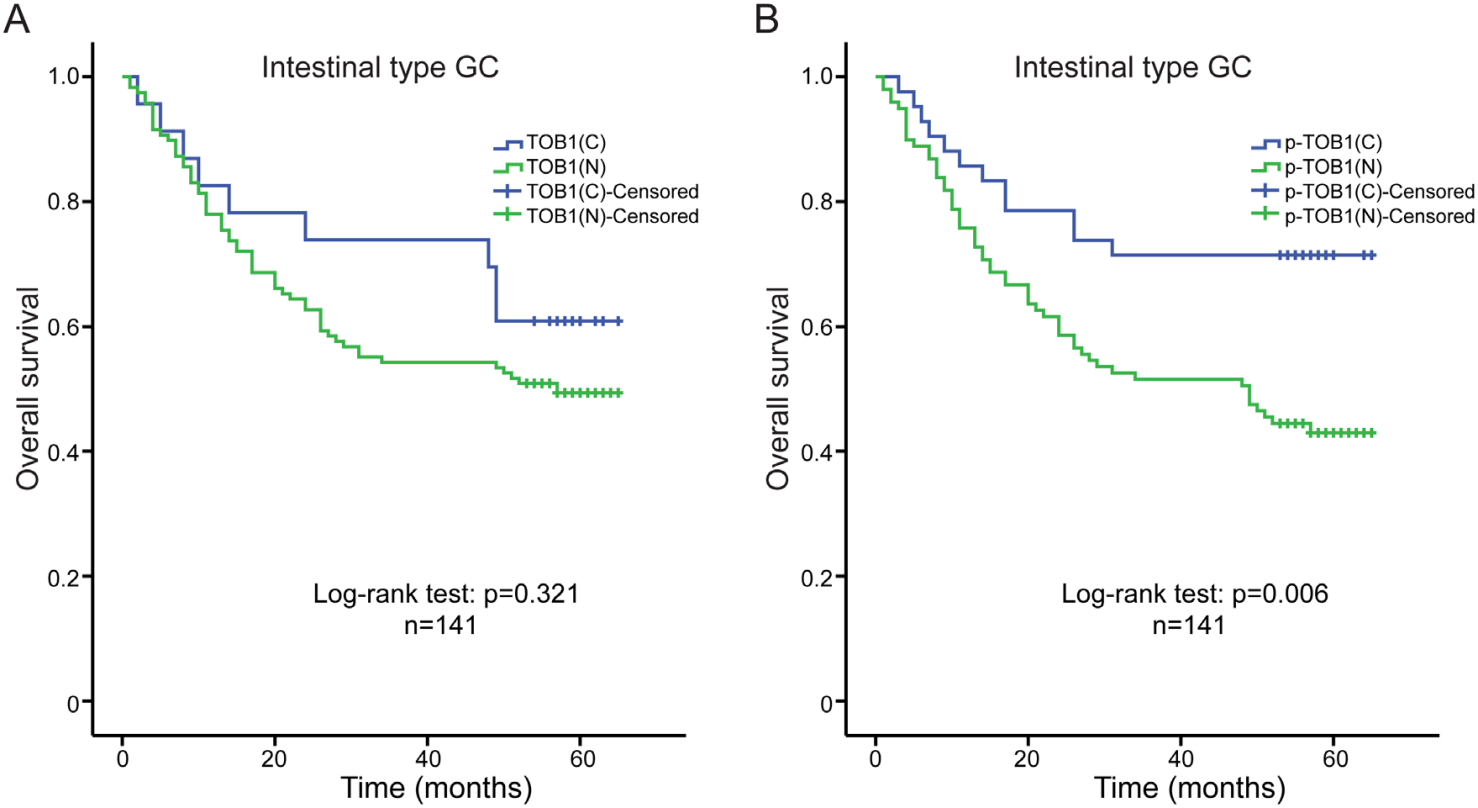
Kaplan-Meier analyses of overall survival in intestinal type GC patients (n = 141) **(A)** Correlation between the subcellular distribution of TOB1 and overall survival. **(B)** Correlation between the subcellular distribution of p-TOB1 with overall survival. C, cytoplasm; N, nucleus.

We used Kaplan-Meier analysis and log-rank tests to analyze the association between the levels of nuclear and cytoplasmic TOB1 or p-TOB1 and survival time in 261 GC patients. No differences in overall survival and TOB1 or p-TOB1 subcellular distribution were observed (data not shown). We also analyzed overall survival in 141 patients with intestinal type and 80 patients with diffuse type GC. Univariate analysis indicated there was no correlation between the subcellular localization of TOB1 (nuclear vs. cytoplasmic) and the overall survival of intestinal type GC patients (log-rank test: *P* = 0.321) (Figure 2A). However, higher nuclear p-TOB1 relative to the cytoplasmic level was associated with worse overall survival in the Kaplan-Meier analysis (log-rank test: *P* = 0.006, Figure 2B). The 5-year cumulative survival rate was 71.4% for patients with low nuclear p-TOB1 expression and 43.4% for those with high nuclear expression of p-TOB1. The mean survival time for patients with low and high nuclear expression of p-TOB1 was 50.5 months and 39.0 months, respectively. Univariate Cox regression analysis showed that patients with low levels of nuclear p-TOB1 had a reduced risk of death (hazard ratio [HR], 0.427; 95% confidence interval [CI], 0.229–0.798; *P* = 0.008) compared to those with high levels of nuclear p-TOB1. This correlation was also observed in a multivariable Cox regression analysis, where nuclear p-TOB1 emerged as an independent prognosticator of reduced overall survival in patients with intestinal type GC (*P* = 0.011, Table 3). These data suggest that high levels of nuclear p-TOB1 is a marker of poor prognosis in patients with intestinal type GC.

**Table 3:** Multivariate analysis of the correlation between clinicopathological characteristics and survival time of intestinal type GC patients

Variables	Multivariate analysis		
	HR	95% CI for HR	*P* value
Lymph node metastases (negative vs. positive)	0.204	0.063-0.666	0.008**
Distant metastasis (negative vs. positive)	0.157	0.044-0.558	0.004**
TNM stage (I-II vs. III-IV)	0.371	0.182-0.755	0.006**
Subcellular distribution of p-TOB1 (C vs. N)	0.420	0.216-0.817	0.011*

CI, confidence interval; HR, hazard ratio; C, cytoplasm; N, nucleus.

### Correlation between TOB1 nuclear localization and anti-proliferative activity

**Figure 3:**
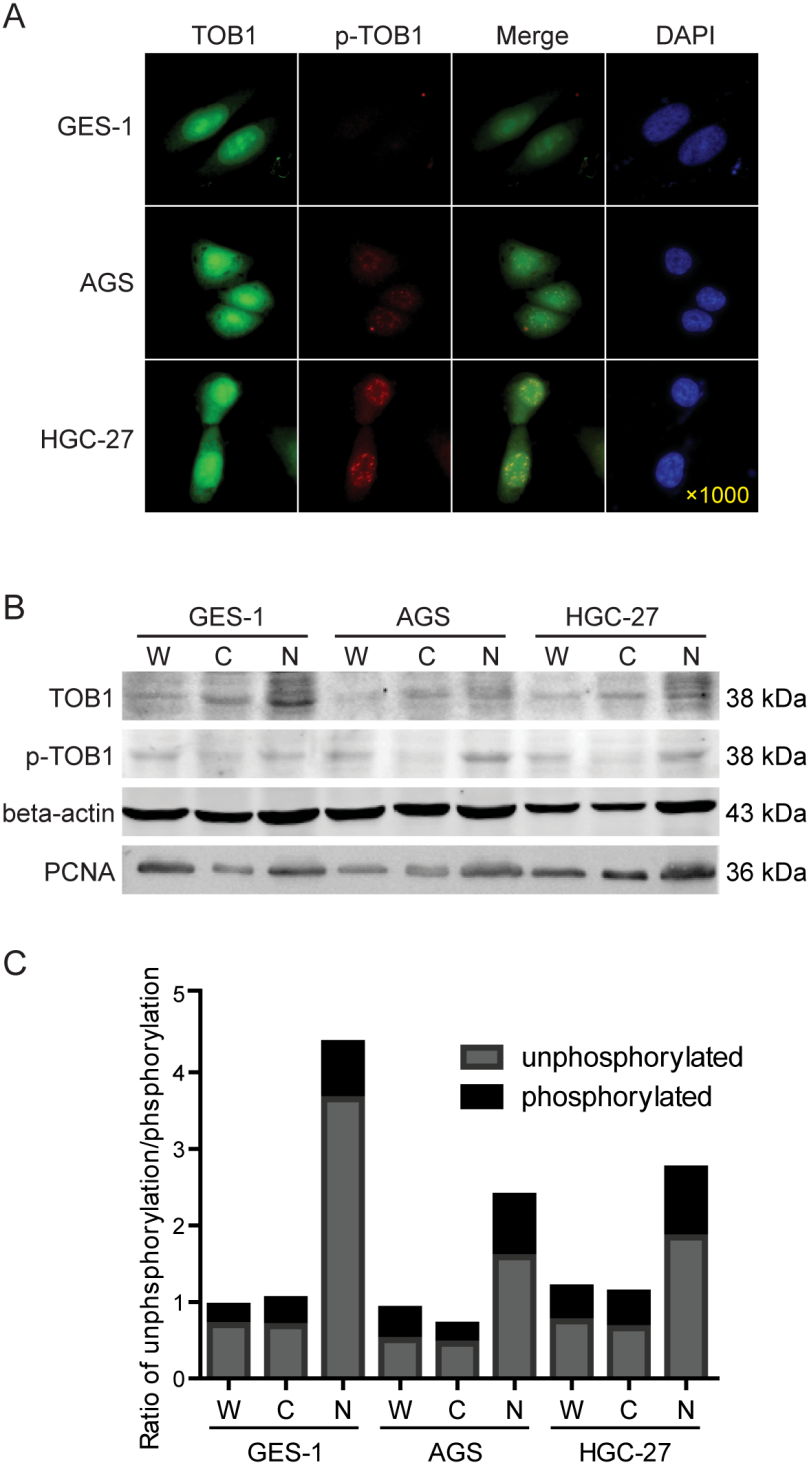
The subcellular distribution of TOB1 and p-TOB1 protein in AGS and HGC-27 GC cells **(A)** Immunofluorescence analysis. **(B)** Western blot analysis of the levels of TOB1 and p-TOB1. **(C)** Ratio of TOB1 to p-TOB1. W, whole cell lysate; C, cytoplasmic fraction; N, nuclear fraction. Cell nuclei were detected with DAPI. GES-1 normal gastric mucosal epithelial cells were used as control.

We analyzed the localization of TOB1 and p-TOB1 in GC cell lines by immunofluorescence. We selected AGS GC cells with low TOB1 expression, HGC-27 cells with high TOB1 expression (unpublished data), and normal GES-1 gastric mucosa cells as a control. Endogenous TOB1 protein was predominantly concentrated in the nuclei of GES-1, AGS, and HGC-27 cells (Figure 3A). However, TOB1 expression was lower in malignant compared to normal gastric mucosa cells. Endogenous p-TOB1 was also primarily detected in the nuclei of AGS and HGC-27 cells. However, little p-TOB1 was present in the nuclei of GES-1 cells. The immunofluorescence results were confirmed by Western blot (Figure 3B and 3C). These data suggest that TOB1 nuclear localization is important for anti-proliferative activity and that accumulation of p-TOB1 promotes GC development.

**Figure 4:**
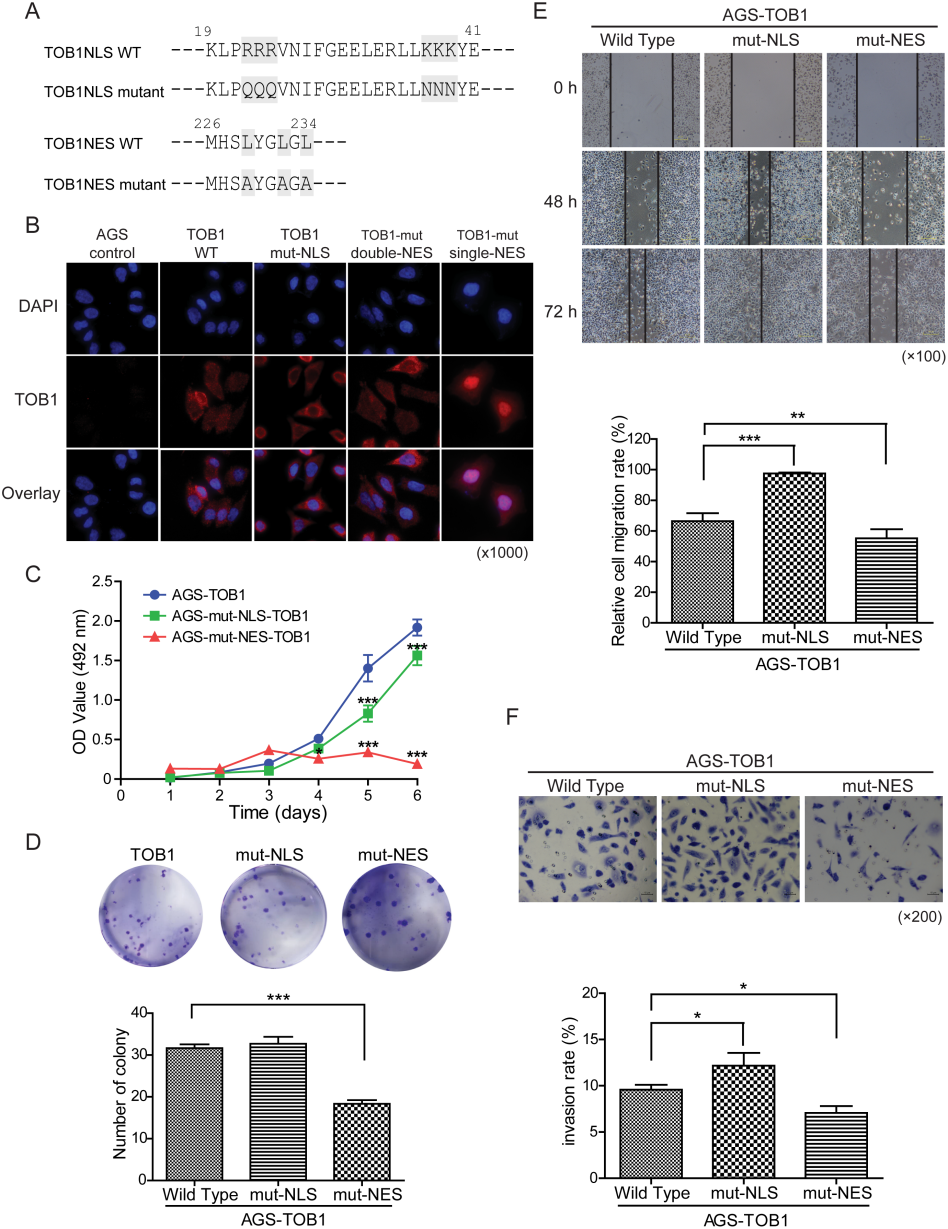
The effects of TOB1 nuclear localization mutants on the malignant potential of AGS cells **(A)** Mutations in the NLS and NES in the TOB1 expression vector. **(B)** Subcellular localization of the mutant TOB1 variants in tumor cells stained with an anti-Flag antibody. **(C)** Growth rate of GC cells. **(D)** Proliferation rate of GC cells. **(E)** Analysis of GC cell migration at the indicated time points following wound formation. Representative graph showing the percentage of cells that migrated relative to the total. **(F)** Analysis of GC cell invasion through Matrigel-coated membranes. Representative images and the percentage of invaded cells are shown.

Because we found that exogenous overexpression of TOB1 suppressed the growth of AGS cells (unpublished data), we analyzed the function of nuclear TOB1 in AGS GC cells. We investigated whether disruption of the nuclear localization of TOB1 affected its anti-proliferative activity in these cells. Kawamura-Tsuzuku et al. showed that trafficking of TOB1 between the nucleus and cytoplasm was mediated by the nuclear localization signal (NLS) (residues 22-39) and nuclear export signal (NES) (residues 226-234) in NIH3T3 cells [15]. We generated constructs in which residues in the TOB1 NLS were mutated. Arg (R) was substituted with Gln (Q) at amino acid positions 22 to 24 and Lys (K) for Asn (N) at positions 37 to 39 (Figure 4A, top row) [15]. We also introduced Leu (L) to Ala (A) substitutions at positions 226 to 234 in the TOB1 NES (Figure 4A, bottom row) [15].

We generated AGS cells that stably overexpressed wild-type TOB1 (AGS-TOB1-WT) and mutant TOB1 (AGS-TOB1NLS/NES-mutants) by transducing TOB1-WT and TOB1-NLS/NES mutants into AGS cells using the lentiviral vector GV358. We examined the subcellular distribution of exogenous, Flag-tagged TOB1 in AGS cells using an anti-Flag antibody. Representative immunofluorescence data are shown in Figure 4B. Compared to AGS control cells expressing endogenous TOB1, exogenously expressed WT TOB1 was primarily distributed in the cytoplasm in the majority of AGS-TOB1-WT cells, although some AGS-TOB1-WT cells had both nuclear and cytoplasmic localization. However, NLS-mutant TOB1 was distributed throughout the cytoplasm in nearly all cells, and NES-mutant TOB1 was concentrated in the nucleus in almost all cells. These results indicate that the NLS and NES are critical for TOB1 nuclear localization in AGS cells.

We examined the effects of nuclear localization of TOB1 on its anti-proliferative activity in AGS cells using MTS, colony formation, migration, and invasion assays. Tumor cell growth was reduced in cells with TOB1 concentrated in the nuclei compared to control cells, in both MTS (Figure 4C) and colony formation assays (Figure 4D). We also found that the migration of cancer cells with TOB1 concentrated in the nuclei was reduced compared to control cells (Figure 4E). This observation was confirmed using Boyden chamber invasion assays, in which nuclear concentration of TOB1 resulted in a decrease in the invasion rate compared to control cells (Figure 4F).

## DISCUSSION

We investigated the expression of TOB1 and p-TOB1 in a cohort of 341 GC patients and found that the subcellular localization of TOB1 and p-TOB1 was associated with specific tumor types and patient survival. We demonstrated an association between the subcellular localization of TOB1 and the malignant potential of GC cells *in vitro*. Immunostaining of TOB1 and p-TOB1 in tissue microarrays revealed reduced nuclear TOB1 levels (80.4%, 274/341) and increased phosphorylation of nuclear TOB1 (66.0%, 225/341) in GC specimens compared to adjacent NG tissue.

Previous studies have indicated that p-TOB1 is the inactive form of the TOB1 protein [18]. We previously demonstrated that TOB1 was either absent or present at low levels in 75% (73/97) of GC samples [11]. Moreover, we observed a general reduction in TOB1 and p-TOB1 protein levels, and the ratio of TOB1 to p-TOB1 protein in four different GC cell lines with various degrees of differentiation. These data suggest that at least two distinct mechanisms are involved in the inactivation of TOB1 in GC that result in reduced TOB1 expression and the accumulation of p-TOB1 in GC cells [11].

We found that decreased nuclear TOB1 was positively correlated with lymph node metastasis, distant metastasis, and TNM stage, but that increased nuclear p-TOB1 was correlated with differentiation grade, depth of invasion, and TNM stage. Zhang et al. demonstrated an association between reduced TOB1 expression in the cytoplasm and clinicopathological characteristics of 90 GC patients including depth of invasion, differentiation grade, and TNM stage. Although nuclear TOB1 expression was also reduced in 45.6% (41/90) of the clinical samples compared to noncancerous tissue, reduced nuclear TOB1 levels were not correlated with specific pathological features [17]. Our data indicated that high levels of p-TOB1 in the nuclei of tumor cells were correlated with poor survival in intestinal type GC patients. Multivariate survival analysis suggested that the nuclear concentration of p-TOB1 was an independent prognostic factor for intestinal type GC.

Decreased TOB1 expression and increased TOB1 phosphorylation has been observed in lung [2] and thyroid [3] cancer. Moreover, decreased TOB1 expression in lung cancer (72%, 31/43), and altered TOB1 phosphorylation in lung adenocarcinoma (76%, 16/21) was shown to be an early event (stage I, 78%, 18/23), even in subjects with bronchial dysplasia (100%, 5/5) [2]. The level of p-TOB1, which was directly linked to tumor size, lymph node metastasis, extra thyroid extension, and the presence of poorly differentiated lesions, was shown to promote progression of papillary carcinoma, particularly in the later phase [3]. Increased p-TOB1 was also associated with poor prognosis in node-negative breast cancer [4].

We examined the expression pattern of TOB1 and p-TOB1 in AGS and HGC-27 cells by immunofluorescence. TOB1 levels were reduced while p-TOB1 levels were elevated in the nuclei of both GC cell lines. Endogenous p-TOB1 was primarily concentrated in the nuclei of AGS and HGC-27 cells, but was only present at low levels in GES-1 control cells, suggesting that the ratio of p-TOB1 to TOB1 contributes to the GC malignant tumor phenotype. The AGS cell line was classified as an intrinsic genomic intestinal subtype [19] with enhanced motility and adhesiveness [20]. The HGC-27 cell line was established from a metastatic lymph node of a GC patient in 1976 [21] and was classified as an intrinsic genomic diffuse subtype associated with Lauren’s histopathology [19].

We investigated the effect of TOB1 subcellular localization on the phenotype of tumor cells by mutating the NLS and NES sequences of TOB1 and stably overexpressing mutant TOB1 in AGS cells *in vitro*. Reduced proliferation, migration, and invasion were observed when TOB1 was concentrated in the nucleus. Kawamura-Tsuzuku et al. suggested that the anti-proliferative activity of TOB1 was correlated with its nuclear localization in NIH3T3 cells [15]. We found that the nuclear localization of TOB1 was essential for anti-proliferative activity in GC cells. Kawamura-Tsuzuku et al. also demonstrated that cytoplasmic TOB1 was increased in ErbB2-transformed NIH3T3 cells, suggesting that impaired nuclear localization promoted phosphorylation of TOB1 and cellular transformation [15].

Phosphorylated TOB1 is inactive in lung and thyroid tumors [2, 3]. We also observed decreased TOB1 and increased p-TOB1 in the nuclei of GC cells from 341 patients with primary GC. We also observed higher cytoplasmic TOB1 (67/341, 19.6%) in GCs and lower cytoplasmic TOB1 (26/341, 7.6%) in NGs (Table 1). It is possible that proliferation was not suppressed in these tumor cells, despite the cytoplasmic localization of TOB1, because TOB1 was phosphorylated by Erk1/2, which is a downstream effector of the ErbB2 receptor [15]. Phosphorylation of TOB1 at specific serine residues (Ser152, Ser154, and Ser164) by Erk1 and Erk2 is required for Ras-mediated cell proliferation and transformation [18].

In summary, we determined that a decrease in TOB1 and an increase in p-TOB1 in the nucleus are key factors in intestinal type GC progression and are associated with a poor prognosis. Nuclear localization of TOB1 is important for its anti-proliferative activity in GC cells.

## MATERIALS AND METHODS

### Tissue specimens

A set of primary GC tissue microarrays (HStm-Ade180CS-01, HStm-Ade180Sur-02, HStm-Ade180Sur-06, and HStm-Ade180Sur-07), which contained 341 pairs of cancerous and noncancerous tissues, was purchased from Shanghai Outdo Biotech Co. Ltd. (Shanghai, China) and used for the immunohistochemistry analysis. None of the patients received pre-operative chemotherapy or radiotherapy. Each GC tissue sample came with detailed patient information including gender, age, stage, and histological type (Supplementary Table 1). All samples were histologically classified as gastric adenocarcinomas, which included 195 cases with intestinal type, 94 with the diffuse type, and 49 with the mixed type according to Lauren’s classification. Survival information was available for 261 of these patients. All work involving human samples was performed in accordance with the Declaration of Helsinki. This project was approved by the Ethics Committee of Harbin Medical University, and informed consent was obtained from all participants.

### Cell lines and culture

The human gastric mucosal epithelial cell line GES-1 was provided by the Laboratory of Medical Genetics, China Medical University (Shenyang, China). The human gastric adenocarcinoma cell line HGC-27 was obtained from the Cell Resources Center of Shanghai Life Sciences, Chinese Academy of Sciences (Shanghai, China). GES-1 cells and HGC-27 cells were grown in RPMI-1640 medium supplemented with 10% fetal bovine serum (FBS). The human gastric adenocarcinoma cell line AGS was purchased from the American Type Culture Collection (Manassas, VA, USA), and the cells were grown in F-12K medium supplemented with 10% FBS. These cell lines were authenticated by Beijing Microread Genetics (Beijing, China) using short tandem repeat (STR) analysis.

### Immunohistochemistry

Immunostaining of TOB1 (with the anti-TOB1 antibody, ab168947, Abcam Company Ltd, Cambridge, MA, USA) and p-TOB1 (with the anti-p-TOB1 antibody, ab78915, Abcam) was performed on 341 paired GC tissues and NG tissues as described [22]. TOB1 expression in the nucleus and/or cytoplasm was analyzed by semi-quantitative assessment of the percentage of marked tumor cells and the staining intensity. TOB1 expression was evaluated by integrating the percentage of positive cells and the staining intensity. Staining intensity was scored as follows: negative (0), weak (1), moderate (2), and high (3). The extent of staining was scored according to the percentage of positive cells in the field: negative (0), 1%–25% (1), 26%–50% (2), 51%–75% (3), and 76%–100% (4). The product of the intensity and extent scores was considered the overall immunohistochemistry score (0–4). The percentage of positive cells and staining intensity scores were multiplied to calculate an immunoreactive score (IRS = SI × PP). An IRS ≤3 or >3 represented negative and positive results, respectively. An IRS of 0–5 was indicative of low expression and an IRS of > 5 was indicative of high expression [23]. An IRS_nucleus_ – IRS_cytoplasm_ ≤ 0 (N ≤ C) was considered cytoplasmic concentration, and an IRS_nucleus_ –IRS_cytoplasm_ > 0 (N > C) was considered nuclear concentration.

We analyzed p-TOB1 immunoreactivity in the nuclei and/or cytoplasm. Cases with an immunoreactive ratio of < 10% were classified as p-TOB1 negative, while the remaining cases were classified as p-TOB1 positive. The positive cases were further classified into two groups: a lower group (10%–50% of cells showing immunoreactivity) and a higher group (> 50% of the cells showing immunoreactivity) [3]. Lower expression in the nucleus and higher expression in the cytoplasm (N < C) was considered cytoplasmic concentration, while higher expression in the nucleus and lower expression in the cytoplasm (N ≥ C) was considered nuclear concentration. The immunostaining results were assessed by two independent pathologists who were blinded to the properties of the samples.

### Immunoblotting

Cells were lysed in RIPA buffer containing protease and phosphatase inhibitors. Lysates were cleared by centrifuging at 10,000 × *g* for 15 minutes and separated by SDS-PAGE. Separated proteins were then transferred onto nitrocellulose membranes. The membranes were blocked with fat-free milk and hybridized with primary antibodies against TOB1 (1:1000, Abcam), p-TOB1 (1:1000, Abcam), Flag (1:1000, F1804, Sigma-Aldrich, USA), β-actin (1:500, ZSGB-BIO Company, Beijing, China), and PCNA (1:200, sc-56, Santa Cruz Biotechnology, Santa Cruz, CA, USA), followed by secondary antibodies (anti-rabbit or anti-mouse antibodies, 1:10,000, Rockland, Limerick, PA, USA). Blots were imaged using an Odyssey Infrared Imaging System (Li-COR, Lincoln, NE, USA).

### Immunofluorescence

Cells were seeded onto coverslips in six-well plates to 50% confluence and fixed in 4% paraformaldehyde. Primary antibodies against TOB1, p-TOB1, and Flag were added to the coverslips and incubated overnight at 4°C, followed by an incubation with anti-mouse or anti-rabbit secondary antibodies. Finally, anti-fade reagent containing 4,6-diamidino-2-phenylindole (DAPI) was added to the slides. Images were acquired using a Leica DM5000B microscope (Leica Microsystems, Solms, Germany).

### Generation of stable AGS cells overexpressing TOB1, TOB1-NLS, and TOB1-NES

AGS cells were infected with either the GV358-TOB1, GV358-TOB1-NLS, or GV358-TOB1-NES lentiviral vector (Genechem Co. Ltd, Shanghai, China). Cells were incubated in the presence of polybrene (5 μg/mL) for 3 days. Cells were fixed 72 h after infection, and protein expression detected by immunostaining.

### Cell viability, colony formation, migration, and invasion assays

Tumor cell viability over the course of 7 days was measured using MTS assays (Promega Corporation, Madison, WI, USA). Colony formation assays were performed by growing the cells for 14 days and staining cell colonies with Giemsa. Cell migration was examined at 0 h, 48 h, and 72 h using scratch assays. The relative migration rate was determined by measuring the average area of the wound gap. Cell invasion was assessed using Boyden chambers (BD Biosciences, San Jose, CA, USA) according to the manufacturer’s instructions. The results are displayed as the percentage of cells that migrated relative to the total number of seeded cells.

### Statistical analysis

Statistical analyses of TMAs were performed using SPSS 17.0 (IBM, Armonk, NY, USA). The relationship between the subcellular distribution of TOB1/p-TOB1 protein and the clinicopathological parameters of GC patients was analyzed using *χ*^2^ or Fisher’s exact tests. Survival curves were generated using Kaplan-Meier analysis, and differences between groups were analyzed using log-rank tests. A univariate Cox proportional hazards model was applied to examine the association between overall survival and the subcellular distribution of TOB1/p-TOB1 as well as other clinical parameters. Stepwise multivariate survival analysis was performed using a Cox proportional hazards model. Variables that were significant in the univariate analysis were included in the model with the Backward Wald method. *ANOVA* and *Dunnett* were used to compare differences in cell viability, colony formation, migration, and invasion between the experimental and control groups. *P < 0.05, **P < 0.01, or ***P < 0.001 were considered statistically significant.

## SUPPLEMENTARY MATERIALS TABLE




