# Supplementary material for: Decreased TOB1 expression and increased phosphorylation of nuclear TOB1 promotes gastric cancer
Source: Oncotarget. 2017 Sep 8;8(43):75243–53. doi: 10.18632/oncotarget.20749 (PMC5650416; doi:10.18632/oncotarget.20749)
Supplement: Supplementary file 1 [file oncotarget-08-75243-s001.pdf]

Decreased TOB1 expression and increased phosphorylation of nuclear TOB1 promotes gastric cancer

SUPPLEMENTARY MATERIALS

Supplementary Table 1: The detailed information of tissue specimens from 341 gastric cancer patients

See Supplementary File 1
