# Supplementary material for: Decreased TOB1 expression and increased phosphorylation of nuclear TOB1 promotes gastric cancer
Source: Oncotarget. 2017 Sep 8;8(43):75243–53. doi: 10.18632/oncotarget.20749 (PMC5650416; doi:10.18632/oncotarget.20749)
Supplement: Supplementary file 2 [file oncotarget-08-75243-s002.doc]

**Supplementary Table 1: The** detailed information of tissue specimens from 341 gastric cancer patients

| **No.** | **Gender** | **Age** | **Grade** | **T** | **N** | **M** | **TNM stage** | **Tumor size (cm)** | **Survival (month)** | **Lauren’s classification** |
| --- | --- | --- | --- | --- | --- | --- | --- | --- | --- | --- |
| 1 | Male | 44 | G1 | T1a | N0 | M0 | ⅠA | 3.5×2.3×0.3 | N/A | Intestinal |
| 2 | Female | 42 | G3 | T1a | N0 | M0 | ⅠA | 2.5×2×0.3 | N/A | Intestinal |
| 3 | Male | 43 | G2 | T1b | N0 | M0 | ⅠA | 4×3×0.5 | N/A | Mixed |
| 4 | Male | 57 | G2 | T2 | N0 | M0 | ⅠB | 3×3×1 | N/A | Intestinal |
| 5 | Male | 64 | G2 | T2 | N0 | M0 | ⅠB | 3.5×2.5×1 | N/A | Intestinal |
| 6 | Male | 65 | G2-G3 | T2 | N0 | M0 | ⅠB | 6×4×2 | N/A | Intestinal |
| 7 | Male | 69 | G2-G3 | T2 | N0 | M0 | ⅠB | 5×4.5×1.5 | N/A | Intestinal |
| 8 | Male | 61 | G2 | T2 | N0 | M0 | ⅠB | 3×1.5×1 | N/A | Intestinal |
| 9 | Male | 58 | G3 | T2 | N0 | M0 | ⅠB | 2×1.5×0.6 | N/A | N/A |
| 10 | Male | 38 | G3 | T2 | N0 | M0 | ⅠB | 5.5×3.5×1 | N/A | Diffuse |
| 11 | Female | 50 | G2 | T2 | N0 | M0 | ⅠB | 5×5×1 | N/A | Diffuse |
| 12 | Female | 58 | G2 | T3 | N0 | M0 | ⅡA | 5×5×2 | N/A | Intestinal |
| 13 | Male | 69 | G2 | T3 | N0 | M0 | ⅡA | 5.5×5×1.1 | N/A | Intestinal |
| 14 | Male | 54 | G2-G3 | T3 | N0 | M0 | ⅡA | 9×7.5×2 | N/A | Intestinal |
| 15 | Male | 67 | G2 | T3 | N0 | M0 | ⅡA | 6×6×2 | N/A | Intestinal |
| 16 | Male | 77 | G2 | T3 | N0 | M0 | ⅡA | 6×5×1 | N/A | Intestinal |
| 17 | Male | 76 | G2-G3 | T3 | N0 | M0 | ⅡA | 5×4×1.5 | N/A | Intestinal |
| 18 | Male | 77 | G2 | T3 | N0 | M0 | ⅡA | 6×4×2 | N/A | Intestinal |
| 19 | Male | 53 | G2-G3 | T3 | N0 | M0 | ⅡA | 7×6×2 | N/A | Intestinal |
| 20 | Male | 53 | G2 | T3 | N0 | M0 | ⅡA | 6×4×1.5 | N/A | Intestinal |
| 21 | Male | 65 | G2-G3 | T3 | N0 | M0 | ⅡA | 6×4×1.5 | N/A | Intestinal |
| 22 | Female | 63 | G3 | T3 | N0 | M0 | ⅡA | 6×5×2 | N/A | Intestinal |
| 23 | Male | 60 | G3 | T3 | N0 | M0 | ⅡA | 7×5×1.5 | N/A | Intestinal |
| 24 | Male | 59 | G3 | T3 | N0 | M0 | ⅡA | 4×3×2 | N/A | Intestinal |
| 25 | N/A | N/A | G2 | T2 | N1 | M0 | ⅡA | 5×4×0.6 | N/A | Intestinal |
| 26 | Female | 47 | G2-G3 | T2 | N1 | M0 | ⅡA | 3×3×1 | N/A | Intestinal |
| 27 | Female | 64 | G2 | T2 | N1 | M0 | ⅡA | 2×2×1 | N/A | Intestinal |
| 28 | Female | 78 | G2 | T4a | N0 | M0 | ⅡB | 2×2×1 | N/A | Intestinal |
| 29 | Male | 61 | G2-G3 | T4a | N0 | M0 | ⅡB | 4×4×2 | N/A | Intestinal |
| 30 | Male | 55 | G1-G2 | T3 | N1 | M0 | ⅡB | 3.5×3.5×2 | N/A | Intestinal |
| 31 | Male | 55 | G2 | T3 | N1 | M0 | ⅡB | 4×2×1 | N/A | Intestinal |
| 32 | Male | 58 | G2 | T3 | N1 | M0 | ⅡB | 5×4×0.7 | N/A | Intestinal |
| 33 | Male | 60 | G2 | T3 | N1 | M0 | ⅡB | 3.6×3.5 | N/A | Intestinal |
| 34 | Female | 51 | G3 | T3 | N1 | M0 | ⅡB | 5×4×2 | N/A | Mixed |
| 35 | Male | 75 | G3 | T3 | N1 | M0 | ⅡB | 4×3×0.6 | N/A | Diffuse |
| 36 | Female | 76 | G2-G3 | T3 | N1 | M0 | ⅡB | 4×4×1.2 | N/A | Diffuse |
| 37 | Female | 50 | G2 | T2 | N2 | M0 | ⅡB | 2×1×0.5 | N/A | Intestinal |
| 38 | Male | 46 | G2-G3 | T2 | N2 | M0 | ⅡB | 3.5×3.5×1 | N/A | Intestinal |
| 39 | Male | 67 | G2 | T3 | N2 | M0 | ⅢA | 8×6×2 | N/A | Intestinal |
| 40 | Male | 60 | G2-G3 | T3 | N2 | M0 | ⅢA | 5×4×1.5 | N/A | Intestinal |
| 41 | Female | 64 | G2-G3 | T3 | N2 | M0 | ⅢA | 4×2×1.5 | N/A | Intestinal |
| 42 | Male | 50 | G2-G3 | T3 | N2 | M0 | ⅢA | 4×4×2.5 | N/A | Intestinal |
| 43 | Male | 63 | G2-G3 | T3 | N2 | M0 | ⅢA | 5×4×4 | N/A | Intestinal |
| 44 | Female | 47 | G3 | T3 | N2 | M0 | ⅢA | 8×7×2 | N/A | Diffuse |
| 45 | Male | 55 | G3 | T3 | N2 | M0 | ⅢA | 5×5×1.5 | N/A | Mixed |
| 46 | Male | 55 | G3 | T3 | N2 | M0 | ⅢA | 4×3.5×2.5 | N/A | Intestinal |
| 47 | Male | 57 | G3 | T2 | N3a | M0 | ⅢA | 4.5×3.5×1.5 | N/A | Intestinal |
| 48 | Male | 57 | G2-G3 | T2 | N3a | M0 | ⅢA | 13×6×2 | N/A | Diffuse |
| 49 | Female | 66 | G3 | T2 | N3a | M0 | ⅢA | 3.5×3×2.5 | N/A | Intestinal |
| 50 | Male | 81 | G2 | T2 | N3a | M0 | ⅢA | 5×2.5×1 | N/A | Intestinal |
| 51 | Female | 63 | G3 | T4b | N0 | M0 | ⅢB | 8×6.5×2 | N/A | Diffuse |
| 52 | Female | 64 | G2-G3 | T4b | N1 | M0 | ⅢB | 5×4×1.3 | N/A | Intestinal |
| 53 | Female | 56 | G2-G3 | T4a | N2 | M0 | ⅢB | 4.5×4×2 | N/A | Mixed |
| 54 | Male | 77 | G3 | T4a | N2 | M0 | ⅢB | 7×7×1.5 | N/A | Mixed |
| 55 | Female | 69 | G2-G3 | T4a | N2 | M0 | ⅢB | 3.5×2.5×1 | N/A | Intestinal |
| 56 | Male | 62 | G2-G3 | T3 | N3a | M0 | ⅢB | 6×5×1 | N/A | Intestinal |
| 57 | Male | 68 | G2 | T3 | N3a | M0 | ⅢB | 2×2×1.5 | N/A | Intestinal |
| 58 | Male | 50 | G2-G3 | T3 | N3a | M0 | ⅢB | 4×3×1 | N/A | Mixed |
| 59 | Male | 67 | G2-G3 | T3 | N3a | M0 | ⅢB | 8×6×3 | N/A | Mixed |
| 60 | Male | 87 | G3 | T3 | N3a | M0 | ⅢB | 6×5×2 | N/A | Diffuse |
| 61 | Male | 56 | G3 | T3 | N3a | M0 | ⅢB | 6×3.5×1.5 | N/A | Intestinal |
| 62 | Male | 69 | G2-G3 | T3 | N3a | M0 | ⅢB | 7×5.5×2 | N/A | Diffuse |
| 63 | Female | 47 | G2-G3 | T3 | N3b | M0 | ⅢB | 3.5×3×1 | N/A | Diffuse |
| 64 | Male | 79 | G2 | T3 | N3b | M0 | ⅢB | 5×4×2 | N/A | Intestinal |
| 65 | Female | 84 | G3 | T3 | N3b | M0 | ⅢB | 7×6×2 | N/A | Mixed |
| 66 | Male | 80 | G2-G3 | T3 | N3b | M0 | ⅢB | 8×7×4 | N/A | Intestinal |
| 67 | Male | 72 | G3 | T4a | N3a | M0 | ⅢC | 8×6×1 | N/A | Mixed |
| 68 | Male | 48 | G3 | T4a | N3a | M0 | ⅢC | 6×5×1 | N/A | Intestinal |
| 69 | Male | 70 | G3 | T4a | N3a | M0 | ⅢC | 6×3.5×1.2 | N/A | Mixed |
| 70 | Male | 75 | G3 | T4b | N2 | M0 | ⅢC | 13×10×2.5 | N/A | Mixed |
| 71 | Male | 65 | G3 | T4b | N3b | M0 | ⅢC | 4.5×3×1 | N/A | Diffuse |
| 72 | Male | 69 | G3 | T3 | N2 | M1 | Ⅳ | 5×4.5×2 | N/A | Intestinal |
| 73 | Male | 57 | G2-G3 | T3 | N2 | M1 | Ⅳ | 6×5.5×2 | N/A | Intestinal |
| 74 | Male | 66 | G3 | T4b | N3a | M1 | Ⅳ | 4.5×3.5×1.5 | N/A | Intestinal |
| 75 | Female | 40 | G3 | N/A | N3a | M1 | Ⅳ | 10×7×3 | N/A | Diffuse |
| 76 | Female | 75 | G3 | T3 | N3a | M1 | Ⅳ | 5.5×4.5×3.5 | N/A | Diffuse |
| 77 | Male | 61 | G2 | N/A | N2 | M1 | Ⅳ | 7×5×1.5 | N/A | Intestinal |
| 78 | Male | 50 | G2-G3 | T3 | N3a | M1 | Ⅳ | 10×8×2.8 | N/A | Intestinal |
| 79 | Male | 70 | G3 | T4b | N3a | M1 | Ⅳ | 4×4×2 | N/A | Diffuse |
| 80 | Male | 62 | G3 | T3 | N0 | M1 | Ⅳ | 4.5×4×3 | N/A | Intestinal |
| 81 | Female | 52 | G3 | T4a | N3b | M0 | ⅢC | 7×5×2 | 3 | Diffuse |
| 82 | Male | 67 | G2-G3 | T3 | N3a | M0 | ⅢB | 10×9×1.6 | 1 | Mixed |
| 83 | Male | 72 | G3 | T3 | N2 | M0 | ⅢA | 7×6×1.5 | 2 | Intestinal |
| 84 | Male | 73 | G3 | T4a | N3b | M0 | ⅢC | 9×5×1.5 | 3 | Diffuse |
| 85 | Female | 42 | G2-G3 | T4b | N2 | M0 | ⅢC | 7×7×1 | 8 | Intestinal |
| 86 | Male | 61 | G3-G4 | T3 | N0 | M0 | ⅡA | 3×3×1 | 65 | Diffuse |
| 87 | Male | 56 | G3 | T3 | N3a | M0 | ⅢB | 3×2×1 | 65 | Diffuse |
| 88 | Female | 54 | G3 | T4b | N2 | M0 | ⅢC | 11×10×1 | 5 | Diffuse |
| 89 | Male | 65 | G2-G3 | T4a | N2 | M0 | ⅢB | 8×6×1 | 65 | Intestinal |
| 90 | Male | 62 | G2 | T1b | N0 | M0 | ⅠA | 4×2.8×0.3 | 65 | Intestinal |
| 91 | Male | 57 | G1 | T1b | N0 | M0 | ⅠA | 3×2×1 | 65 | Intestinal |
| 92 | Female | 79 | G2-G3 | T3 | N0 | M0 | ⅡA | 5.5×4×1 | 17 | Intestinal |
| 93 | Female | 71 | G2-G3 | T2 | N1 | M0 | ⅡA | 4×3×1 | 65 | Mixed |
| 94 | Male | 57 | G3 | T3 | N3a | M0 | ⅢB | 5×4×1 | 65 | Diffuse |
| 95 | Male | 68 | G3 | T4a | N3a | M0 | ⅢC | 9×6×1 | 8 | Diffuse |
| 96 | Male | 53 | G3 | T3 | N1 | M0 | ⅡB | 4.5×3.5×1 | 17 | Intestinal |
| 97 | Male | 71 | G2-G3 | T4a | N2 | M0 | ⅢB | 9×5.5×1 | 10 | Intestinal |
| 98 | Female | 68 | G2-G3 | T4a | N1 | M0 | ⅢA | 4×4×1.5 | 5 | Intestinal |
| 99 | Male | 61 | G3 | T4a | N3a | M0 | ⅢC | 13×5.5×2 | 0 | Diffuse |
| 100 | Female | 68 | G2 | T3 | N1 | M0 | ⅡB | 7.5×5.5×2 | 11 | Intestinal |
| 101 | Female | 65 | G3 | T3 | N0 | M0 | ⅡA | 4×2.5×1.5 | 65 | Intestinal |
| 102 | Male | 72 | G3 | T2 | N2 | M1 | Ⅳ | 4.5×4×1.5 | 6 | Intestinal |
| 103 | Male | 49 | G3-G4 | T3 | N0 | M0 | ⅡA | 5×4×1.5 | 1 | Diffuse |
| 104 | Male | 50 | G2-G3 | T4a | N2 | M0 | ⅢB | 7.5×3.5×1.5 | 65 | Intestinal |
| 105 | Female | 52 | G3-G4 | T3 | N3b | M0 | ⅢB | 6×3×1.5 | 16 | Diffuse |
| 106 | Male | 78 | G3 | T3 | N3b | M0 | ⅢB | 15×9×1.5 | 8 | Intestinal |
| 107 | Male | 67 | G3 | T3 | N0 | M0 | ⅡA | 10×10×1.5 | 65 | Intestinal |
| 108 | Male | 75 | G2-G3 | T4b | N3a | M0 | ⅢC | 6×4×1 | 0 | Mixed |
| 109 | Male | 65 | G3 | T3 | N0 | M0 | ⅡA | 6×4×1 | 3 | Diffuse |
| 110 | Male | 75 | G3 | T4a | N3b | M0 | ⅢC | 6×6×2 | 7 | Diffuse |
| 111 | Male | 78 | G3 | T3 | N0 | M0 | ⅡA | 8×8×5 | 64 | Diffuse |
| 112 | Male | 51 | G3 | T3 | N2 | M0 | ⅢA | 7×4×1.5 | 10 | Diffuse |
| 113 | Male | 74 | G2-G3 | T4a | N2 | M0 | ⅢB | 2.8×2×1 | 64 | Intestinal |
| 114 | Female | 67 | G3 | T3 | N3a | M0 | ⅢB | 5×4×2 | 4 | Diffuse |
| 115 | Male | 55 | G3 | T3 | N2 | M0 | ⅢA | 8×7×1.5 | 64 | Mixed |
| 116 | Male | 62 | G3 | T3 | N0 | M0 | ⅡA | 7.5×5×2.5 | 12 | Mixed |
| 117 | Male | 66 | G3 | T4a | N3a | M0 | ⅢC | 7×5×1 | 2 | Mixed |
| 118 | Male | 72 | G3-G4 | T3 | N0 | M0 | ⅡA | 4×2.5×1 | 64 | Diffuse |
| 119 | Male | 67 | G2-G3 | T3 | N0 | M0 | ⅡA | 3.5×3×1.5 | 64 | Intestinal |
| 120 | Male | 73 | G2 | T3 | N2 | M0 | ⅢA | 3.5×3.5×1 | 26 | Intestinal |
| 121 | Female | 80 | G3 | T3 | N0 | M0 | ⅡA | 7×4.5×1.5 | 64 | Intestinal |
| 122 | Male | 59 | G3 | T4a | N2 | M0 | ⅢB | 3×2×1.5 | 54 | Diffuse |
| 123 | Male | 74 | G2 | T4a | N0 | M0 | ⅡB | 5×4×1 | 63 | Intestinal |
| 124 | Female | 51 | G3 | T3 | N0 | M0 | ⅡA | 3.5×2×1 | 63 | Diffuse |
| 125 | Female | 76 | G3 | T3 | N3a | M0 | ⅢB | 8×7×1 | 30 | Diffuse |
| 126 | Male | 64 | G2 | T2 | N1 | M0 | ⅡA | 7×5×0.5 | 63 | Intestinal |
| 127 | Male | 52 | G3-G4 | T3 | N1 | M0 | ⅡB | 4×3×2;2×2×1;2×1.5×1 | 22 | Diffuse |
| 128 | Male | 54 | G3 | T3 | N3a | M0 | ⅢB | 3×3×1 | 19 | Mixed |
| 129 | Male | 58 | G3 | T3 | N0 | M0 | ⅡA | 6×5×1.5 | 62 | Intestinal |
| 130 | Male | 63 | G3 | T3 | N3b | M0 | ⅢB | 15×11×1 | 15 | Diffuse |
| 131 | Male | 67 | G3 | T3 | N0 | M0 | ⅡA | 3.5×1.5×1 | 62 | Mixed |
| 132 | Female | 65 | G3 | T3 | N3b | M0 | ⅢB | 5×4×1 | 28 | Diffuse |
| 133 | Male | 55 | G3-G4 | T4b | N3a | M0 | ⅢC | 6×4×1.5 | 29 | Diffuse |
| 134 | Male | 68 | G2-G3 | T2 | N0 | M0 | ⅠB | 4.5×3.5×1.5 | 62 | Intestinal |
| 135 | Male | 80 | G3 | T3 | N3a | M0 | ⅢB | 7.5×4.5×1.5 | 10 | Intestinal |
| 136 | Male | 54 | G3-G4 | T3 | N0 | M0 | ⅡA | 5.5×3×1.5 | 62 | Mixed |
| 137 | Male | 54 | G2 | T3 | N2 | M0 | ⅢA | 10×9×2 | 15 | Intestinal |
| 138 | Male | 72 | G3 | T3 | N1 | M0 | ⅡB | 9×6×2 | 22 | Intestinal |
| 139 | Male | 72 | G3 | T3 | N3a | M0 | ⅢB | 5.5×5×1 | 61 | Mixed |
| 140 | Female | 46 | G1-G2 | T1b | N0 | M0 | ⅠA | 4×2.5×0.5 | 61 | Intestinal |
| 141 | Female | 74 | G3-G4 | T3 | N3a | M0 | ⅢB | 27×12×1.3 | 8 | Diffuse |
| 142 | Female | 60 | G3 | T4a | N2 | M0 | ⅢB | 9×6×1 | 17 | Mixed |
| 143 | Male | 68 | G3 | T3 | N2 | M0 | ⅢA | 5.5×5×1.5 | 18 | Diffuse |
| 144 | Female | 68 | G3-G4 | T3 | N0 | M0 | ⅡA | 20×13×1.5 | 54 | Diffuse |
| 145 | Male | 61 | G2 | T3 | N2 | M0 | ⅢA | 7×5×1.5 | 50 | Intestinal |
| 146 | Female | 61 | G3 | T3 | N2 | M0 | ⅢA | 7×6.5×2 | 6 | Diffuse |
| 147 | Female | 77 | G2-G3 | T4a | N2 | M0 | ⅢB | 5×5×1 | 61 | Intestinal |
| 148 | Male | 68 | G3 | T3 | N0 | M0 | ⅡA | 3×3×1 | 61 | Intestinal |
| 149 | Male | 56 | G3-G4 | T3 | N3a | M0 | ⅢB | 5×3×1 | 61 | Diffuse |
| 150 | Female | 70 | G3 | T2 | N1 | M0 | ⅡA | 2×1.5×0.5 | 22 | Mixed |
| 151 | Female | 78 | G2-G3 | T4a | N2 | M0 | ⅢB | 5.5×4×1.3 | 61 | Mixed |
| 152 | Female | 75 | G3 | T3 | N3a | M0 | ⅢB | 4×2.5×1.5 | 24 | Diffuse |
| 153 | Male | 76 | G2-G3 | T4a | N3b | M0 | ⅢC | 3.5×2.5×1.5 | 0 | Diffuse |
| 154 | Male | 68 | G3 | T3 | N2 | M0 | ⅢA | 7×6×1.5 | 34 | Intestinal |
| 155 | Male | 77 | G3 | T2 | N2 | M0 | ⅡB | 4×3×1 | 22 | Mixed |
| 156 | Male | 57 | G2-G3 | T3 | N3a | M0 | ⅢB | 4×3×1 | 15 | Intestinal |
| 157 | Male | 68 | G2 | T3 | N2 | M0 | ⅢA | 5×4×1.5 | 13 | Intestinal |
| 158 | Female | 52 | G3 | T4a | N3b | M0 | ⅢC | 2.5×2×1 | 27 | Diffuse |
| 159 | Female | 52 | G2 | T3 | N1 | M0 | ⅡB | 6×5×2 | 60 | Intestinal |
| 160 | Female | 82 | G3 | T3 | N2 | M0 | ⅢA | 5×5×1.5 | 7 | Diffuse |
| 161 | Female | 51 | G3 | T3 | N3b | M0 | ⅢB | 12×11×1.5 | 24 | Diffuse |
| 162 | Male | 57 | G3 | T4a | N2 | M0 | ⅢB | 4×4×1 | 60 | Intestinal |
| 163 | Male | 69 | G2 | T3 | N0 | M0 | ⅡA | 8×7×2.5 | 60 | Intestinal |
| 164 | Female | 51 | G3 | T3 | N2 | M0 | ⅢA | 10×9×1.5 | 24 | Diffuse |
| 165 | Male | 60 | G3 | T3 | N3b | M0 | ⅢB | 5×5×1 | 24 | Diffuse |
| 166 | Female | 79 | G2-G3 | T4a | N3a | M0 | ⅢC | 3×2.5×1.5 | 8 | Intestinal |
| 167 | Male | 71 | G2-G3 | T3 | N0 | M0 | ⅡA | 4×4×2 | 60 | Intestinal |
| 168 | Female | 31 | G3 | T4b | N3b | M0 | ⅢC | 6×5×4 | 12 | Diffuse |
| 169 | Male | 61 | G3 | T3 | N3a | M1 | Ⅳ | 4×2×1 | 15 | Else |
| 170 | Male | 55 | G2 | T3 | N3a | M0 | ⅢB | 5×4×1.5 | 1 | Intestinal |
| 171 | Male | 76 | G2-G3 | T3 | N3a | M0 | ⅢB | 5×3×1.5 | 20 | Intestinal |
| 172 | Male | 64 | G3 | T4a | N3a | M0 | ⅢC | 8×6×2.5 | 12 | Diffuse |
| 173 | Male | 51 | G3 | T4a | N3b | M0 | ⅢC | 12×18×1.5 | 22 | Diffuse |
| 174 | Male | 76 | G2-G3 | T3 | N3a | M0 | ⅢB | 12×8×1 | 24 | Intestinal |
| 175 | Female | 45 | G3 | T4a | N3a | M0 | ⅢC | 3×1.5×1 | 16 | Diffuse |
| 176 | Male | 69 | G2 | T2 | N0 | M0 | ⅠB | 4×3×0.5 | 59 | Intestinal |
| 177 | Female | 79 | G2 | T2 | N1 | M0 | ⅡA | 2×1×1 | 59 | Intestinal |
| 178 | Male | 54 | G3 | T3 | N3a | M0 | ⅢB | 7×6×2 | 46 | Diffuse |
| 179 | Female | 45 | G3 | T3 | N3a | M0 | ⅢB | 2×2×0.8 | 59 | Mixed |
| 180 | Male | 52 | G3 | T3 | N3a | M0 | ⅢB | 7×4×1.5 | 13 | Intestinal |
| 181 | Female | 75 | G3 | T2 | N0 | M0 | ⅠB | 3×3×1 | 59 | Intestinal |
| 182 | Female | 33 | G3 | T3 | N1 | M0 | ⅡB | 3×2×1.5 | 59 | Diffuse |
| 183 | Female | 48 | G3 | T3 | N3b | M0 | ⅢB | 6×4.5×1 | 1 | Mixed |
| 184 | Male | 51 | G2 | T3 | N0 | M0 | ⅡA | 8.5×7×2 | 59 | Intestinal |
| 185 | Male | 65 | G2-G3 | T4a | N3b | M0 | ⅢC | 16×4×2 | 4 | Intestinal |
| 186 | Female | 81 | G2-G3 | T3 | N2 | M0 | ⅢA | 9×6×1.5 | 4 | Intestinal |
| 187 | Female | 84 | G2-G3 | T3 | N1 | M0 | ⅡB | 6×5.5×1 | 59 | Intestinal |
| 188 | Male | 61 | G3 | T3 | N3a | M1 | Ⅳ | 4.5×3×1 | 4 | Intestinal |
| 189 | Male | 60 | G3 | T4a | N2 | M0 | ⅢB | 10×9×1 | 7 | Intestinal |
| 190 | Male | 54 | G3 | T4a | N2 | M0 | ⅢB | 5×5×1.5 | 31 | Mixed |
| 191 | Female | 35 | G3 | T3 | N2 | M0 | ⅢA | 2.5×2×1 | 21 | Intestinal |
| 192 | Male | 55 | G3 | T3 | N1 | M0 | ⅡB | 9.5×7×1.5 | 20 | Intestinal |
| 193 | Male | 67 | G2-G3 | T3 | N1 | M0 | ⅡB | 5.5×5×1 | 57 | Intestinal |
| 194 | Female | 74 | G2 | T3 | N3a | M0 | ⅢB | 9×5.5×1.5 | 17 | Intestinal |
| 195 | Male | 70 | G3 | T3 | N0 | M0 | ⅡA | 6×6×3.5 | 57 | N/A |
| 196 | Female | 80 | G1-G2 | T3 | N1 | M0 | ⅡB | 3×3×0.5 | 57 | Intestinal |
| 197 | Male | 69 | G2-G3 | T4a | N2 | M0 | ⅢB | 6×6×1.5;1.5×1.5×0.7 | 13 | Intestinal |
| 198 | Female | 74 | G3 | T3 | N1 | M0 | ⅡB | 9×8×1 | 57 | Intestinal |
| 199 | Male | 64 | G2 | T4b | N3a | M0 | ⅢC | 5×3×2 | 4 | Intestinal |
| 200 | Female | 56 | G3 | T3 | N3a | M0 | ⅢB | 5×3.5×2 | 4 | Intestinal |
| 201 | Male | 52 | G3 | T3 | N0 | M0 | ⅡA | 4×3×1 | 57 | Diffuse |
| 202 | Female | 63 | G3 | T3 | N2 | M0 | ⅢA | 7.5×5.5×2.5 | 8 | Diffuse |
| 203 | Male | 59 | G3 | T3 | N0 | M0 | ⅡA | 7.5×3.5×1 | 57 | Diffuse |
| 204 | Female | 66 | G3 | T4a | N3a | M0 | ⅢC | 5×4.5×2 | 21 | Diffuse |
| 205 | Male | 53 | G3 | T3 | N0 | M0 | ⅡA | 6×6×2 | 17 | Intestinal |
| 206 | Female | 54 | G2 | T3 | N0 | M0 | ⅡA | 7×6×1 | 57 | Intestinal |
| 207 | Female | 70 | G2 | T1b | N0 | M0 | ⅠA | 3×2×0.2 | 57 | Intestinal |
| 208 | Male | 60 | G3 | T3 | N0 | M0 | ⅡA | 3.5×3.5×3 | 28 | Mixed |
| 209 | Male | 48 | G3 | T4a | N0 | M0 | ⅡB | 3×2.5×1 | 56 | Diffuse |
| 210 | Male | 71 | G2 | T2 | N3a | M0 | ⅢA | 3×2.5×0.6 | 11 | Intestinal |
| 211 | Male | 62 | G2 | T2 | N0 | M0 | ⅠB | 9×6×1.5 | 56 | Intestinal |
| 212 | Female | 76 | G2 | T3 | N0 | M0 | ⅡA | 5×3.5×1 | 56 | Intestinal |
| 213 | Male | 51 | G3 | T3 | N2 | M0 | ⅢA | 3×3×2 | 5 | Mixed |
| 214 | Male | 70 | G3 | T4a | N2 | M0 | ⅢB | 4×3×1.2 | 53 | Diffuse |
| 215 | Male | 77 | G2-G3 | T2 | N0 | M0 | ⅠB | 5×3×0.7 | 56 | Intestinal |
| 216 | Female | 75 | G2 | T3 | N0 | M0 | ⅡA | 5.5×5×2 | 56 | Intestinal |
| 217 | Male | 63 | G3 | T4a | N1 | M0 | ⅢA | 5.5×5.5×1.5 | 56 | Intestinal |
| 218 | Male | 59 | G3 | T3 | N1 | M0 | ⅡB | 4×3×1 | 7 | Diffuse |
| 219 | Male | 57 | G3 | T2 | N0 | M0 | ⅠB | 3.5×3×1.2 | 56 | Intestinal |
| 220 | Female | 42 | G3 | T3 | N0 | M0 | ⅡA | 3×2×0.5 | 19 | Diffuse |
| 221 | Male | 63 | G2 | T3 | N0 | M0 | ⅡA | 8×6×2;2×1.5×0.5 | 55 | Intestinal |
| 222 | Female | 46 | G3 | T4a | N2 | M0 | ⅢB | 5×4×1 | 46 | Diffuse |
| 223 | Female | 62 | G3 | T2 | N2 | M0 | ⅡB | 7×6.5×1 | 50 | Mixed |
| 224 | Male | 57 | G2 | T4a | N2 | M0 | ⅢB | 5×4.5×2 | 29 | Intestinal |
| 225 | Male | 71 | G3 | T3 | N1 | M0 | ⅡB | 3×2×1 | 55 | Intestinal |
| 226 | Female | 85 | G3 | T4a | N3a | M0 | ⅢC | 13×8×1 | 3 | Intestinal |
| 227 | Female | 48 | G2 | T3 | N3b | M0 | ⅢB | 5.5×4.5×2 | 9 | Intestinal |
| 228 | Male | 56 | G2 | T1b | N1 | M0 | ⅠB | 3.5×3×0.4 | 49 | Intestinal |
| 229 | Female | 28 | G3 | T4a | N2 | M0 | ⅢB | 7.5×7×1 | 14 | Diffuse |
| 230 | Male | 62 | G2 | T1b | N0 | M0 | ⅠA | 3×3×0.5 | 54 | Intestinal |
| 231 | Male | 77 | G3 | T3 | N3b | M0 | ⅢB | 7×5×1 | 1 | Intestinal |
| 232 | Female | 75 | G3 | T3 | N3a | M0 | ⅢB | 7×6×1.5 | 5 | Intestinal |
| 233 | Male | 65 | G2-G3 | T3 | N0 | M0 | ⅡA | 6×4×1.5 | 54 | Intestinal |
| 234 | Male | 53 | G2-G3 | T3 | N3b | M0 | ⅢB | 4×3×1.5 | 4 | Mixed |
| 235 | Male | 59 | G2-G3 | T4b | N3a | M1 | Ⅳ | 7×6×1.7 | 14 | Intestinal |
| 236 | Male | 65 | G2 | T4a | N2 | M0 | ⅢB | 3×2×1.5 | 54 | Intestinal |
| 237 | Male | 47 | G2-G3 | T3 | N0 | M0 | ⅡA | 5×4×1.5 | 54 | Intestinal |
| 238 | Female | 67 | G3 | T3 | N3a | M0 | ⅢB | 3×3×2 | 31 | Mixed |
| 239 | Male | 61 | G3 | T3 | N3b | M0 | ⅢB | 9×4×1 | 13 | Diffuse |
| 240 | Male | 54 | G3 | T3 | N3a | M0 | ⅢB | 3×2×1.2 | 21 | Diffuse |
| 241 | Male | 70 | G2-G3 | T2 | N2 | M0 | ⅡB | 4.5×3×0.8 | 53 | Intestinal |
| 242 | Female | 76 | G2-G3 | T3 | N3b | M0 | ⅢB | 5×3×2.5 | 2 | Intestinal |
| 243 | Female | 66 | G3 | T2 | N3a | M0 | ⅢA | 3.5×3×2.5 | 24 | Intestinal |
| 244 | Male | 64 | G2 | T2 | N0 | M0 | ⅠB | 3.5×2.5×1 | 53 | Intestinal |
| 245 | Male | 88 | G3 | T3 | N2 | M0 | ⅢA | 8×6×0.7 | 7 | Mixed |
| 246 | Female | 69 | G3 | T3 | N3a | M0 | ⅢB | 7.5×5×1 | 12 | Mixed |
| 247 | Female | 47 | G3 | T4a | N3a | M1 | Ⅳ | 10×6×1.3 | 4 | Diffuse |
| 248 | Male | 63 | G3 | T3 | N2 | M0 | ⅢA | 6×6×2.5 | 53 | Intestinal |
| 249 | Female | 59 | G2-G3 | T3 | N3a | M0 | ⅢB | 8×7×1.7 | 11 | Intestinal |
| 250 | Female | 72 | G2 | T4a | N3a | M0 | ⅢC | 5.5×4.5×2 | 49 | Intestinal |
| 251 | Female | 77 | G2-G3 | T4a | N3a | M0 | ⅢC | 3×3×1 | 7 | Intestinal |
| 252 | Male | 73 | G2-G3 | T3 | N2 | M0 | ⅢA | 3×2×1 | 53 | Intestinal |
| 253 | Female | 86 | G2 | T3 | N2 | M0 | ⅢA | 3×3×1 | 27 | Intestinal |
| 254 | Male | 38 | G3 | T2 | N0 | M0 | ⅠB | 5.5×3.5×1 | 59 | Intestinal |
| 255 | Male | 74 | G3 | T3 | N3a | M0 | ⅢB | 4.5×3×1 | 11 | Intestinal |
| 256 | Female | 66 | G3 | T4a | N3a | M1 | Ⅳ | 11×6.5×2.5 | 15 | Diffuse |
| 257 | Female | 75 | G3 | T3 | N3a | M0 | ⅢB | 10×8×1.5 | 54 | Diffuse |
| 258 | Male | 58 | G2-G3 | T4a | N3a | M0 | ⅢC | 8×6×2.5 | 21 | Mixed |
| 259 | Male | 73 | G2 | T3 | N2 | M0 | ⅢA | 4.5×4.5×1 | 59 | Intestinal |
| 260 | Male | 61 | G2-G3 | T2 | N0 | M0 | ⅠB | 3×1.5×1 | 26 | Intestinal |
| 261 | Female | 69 | G2 | T2 | N1 | M0 | ⅡA | 5×4×0.5 | 59 | Intestinal |
| 262 | Female | 50 | G3 | T3 | N2 | M0 | ⅢA | 10×4×1 | 23 | Diffuse |
| 263 | Male | 65 | G2-G3 | T2 | N1 | M0 | ⅡA | 4×3×2.5 | 20 | Intestinal |
| 264 | Male | 58 | G3-G4 | T3 | N3b | M0 | ⅢB | 5×5×1.3 | 8 | Diffuse |
| 265 | Male | 48 | G3 | T4a | N1 | M0 | ⅢA | 5.5×4×1.5 | 58 | Diffuse |
| 266 | Male | 50 | G3 | T3 | N3a | M0 | ⅢB | 5×2×1.5 | 6 | Mixed |
| 267 | Female | 79 | G3 | T2 | N0 | M0 | ⅠB | 2×1.5×1 | 58 | Diffuse |
| 268 | Male | 66 | G2-G3 | T4a | N3b | M0 | ⅢC | 12×6.5×1.5 | 14 | Intestinal |
| 269 | Male | 58 | G3 | T3 | N0 | M0 | ⅡA | 3×2.5×1 | 58 | Intestinal |
| 270 | Male | 62 | G3 | T3 | N3a | M0 | ⅢB | 7×4×1 | 28 | Intestinal |
| 271 | Male | 49 | G2 | T1a | N0 | M0 | ⅠA | 4×3×0.5 | 58 | Intestinal |
| 272 | Male | 68 | G2 | T4a | N3a | M0 | ⅢC | 3×3×1.5 | 58 | Intestinal |
| 273 | Male | 81 | G2 | T2 | N3a | M0 | ⅢA | 5×2.5×1 | 58 | Intestinal |
| 274 | Female | 69 | G3 | T2 | N1 | M0 | ⅡA | 2.5×2×0.7 | 58 | Intestinal |
| 275 | Male | 79 | G2 | T1 | N0 | M0 | ⅠA | 2×2×0.7 | 58 | Intestinal |
| 276 | Male | 62 | G3 | T2 | N0 | M0 | ⅠB | 4.5×4×1.3 | 57 | Intestinal |
| 277 | Male | 83 | G2-G3 | T4a | N0 | M0 | ⅡB | 4×3×1.3 | 57 | Intestinal |
| 278 | Male | 54 | G2 | T3 | N0 | M0 | ⅡA | 3×3×1 | 57 | Intestinal |
| 279 | Male | 77 | G3 | T4a | N2 | M0 | ⅢB | 7×7×1.5 | 26 | Intestinal |
| 280 | Male | 65 | G3 | T3 | N2 | M0 | ⅢA | 6×4×0.6 | 3 | Intestinal |
| 281 | Male | 73 | G2 | T4a | N0 | M0 | ⅡB | 4×3×1 | 57 | Intestinal |
| 282 | Female | 78 | G2 | T2 | N0 | M0 | ⅠB | 3.5×3×1 | 52 | Intestinal |
| 283 | Male | 54 | G2-G3 | T3 | N0 | M0 | ⅡA | 5×4×1 | 57 | Intestinal |
| 284 | Female | 58 | G3 | T3 | N2 | M0 | ⅢA | 4×4×1 | 57 | Diffuse |
| 285 | Female | 64 | G2 | T2 | N1 | M0 | ⅡA | 2×2×1 | 26 | Intestinal |
| 286 | Female | 58 | G3 | T3 | N2 | M0 | ⅢA | 8×7×1 | 57 | Diffuse |
| 287 | Female | 77 | G2 | T3 | N3a | M0 | ⅢB | 6×5×3 | 51 | Intestinal |
| 288 | Female | 73 | G3 | T3 | N2 | M0 | ⅢA | 3×2.7×1.5 | 7 | Mixed |
| 289 | Male | 72 | G3 | T3 | N1 | M0 | ⅡB | 7×5.5×2 | 13 | Mixed |
| 290 | Male | 53 | G2 | T1b | N0 | M0 | ⅠA | 3×2.5×0.7 | 57 | Intestinal |
| 291 | Female | 56 | G3 | T3 | N0 | M0 | ⅡA | 6×3.5×1 | 57 | Mixed |
| 292 | Male | 58 | G3 | T4a | N2 | M0 | ⅢB | 3.5×3×1 | 56 | Mixed |
| 293 | Female | 63 | G2 | T3 | N1 | M0 | ⅡB | 3×3×1.5 | 56 | Intestinal |
| 294 | Male | 55 | G3 | T4b | N3a | M0 | ⅢC | 7×5×1 | 19 | Diffuse |
| 295 | Male | 56 | G3 | T3 | N1 | M0 | ⅡB | 5×5×1 | 22 | Diffuse |
| 296 | Female | 73 | G3 | T3 | N1 | M0 | ⅡB | 6×5×1.5 | 15 | Diffuse |
| 297 | Male | 89 | G3 | T4a | N2 | M0 | ⅢB | 9×8×1 | 17 | Diffuse |
| 298 | Male | 62 | G3 | T4a | N3b | M0 | ⅢC | 4×3×0.5 | 51 | Diffuse |
| 299 | Male | 85 | G3 | T4a | N3a | M0 | ⅢC | 3.5×2×1 | 10 | Diffuse |
| 300 | N/A | 58 | G3 | T4a | N1 | M0 | ⅢA | 4×4×1 | 56 | Diffuse |
| 301 | Male | 32 | G3 | T3 | N2 | M0 | ⅢA | 3×2.5×1.5 | 56 | Diffuse |
| 302 | Male | 58 | G2 | T3 | N2 | M0 | ⅢA | 9×6×1.5 | 55 | Intestinal |
| 303 | Male | 69 | G3 | T3 | N2 | M0 | ⅢA | 5×5×0.8 | 24 | Mixed |
| 304 | Male | 63 | G3 | T3 | N3a | M0 | ⅢB | 6×6×1 | 10 | Intestinal |
| 305 | Male | 71 | G3 | T3 | N3a | M0 | ⅢB | 5×5×1.5 | 55 | Intestinal |
| 306 | Female | 85 | G3 | T4a | N1 | M0 | ⅢA | 6.5×6×1 | 55 | Intestinal |
| 307 | Female | 52 | G3 | T4a | N2 | M0 | ⅢB | 14×10×1 | 55 | Mixed |
| 308 | Male | 69 | G2-G3 | T4a | N2 | M0 | ⅢB | 6×5×1.5 | 31 | Intestinal |
| 309 | Male | 53 | G3 | T4a | N1 | M0 | ⅢA | 6×4×1 | 55 | Diffuse |
| 310 | Male | 46 | G3 | T4a | N0 | M0 | ⅡB | 3.5×2×1 | 18 | Diffuse |
| 311 | Male | 58 | G3 | T3 | N3b | M0 | ⅢB | 6×6×1.5 | 31 | Intestinal |
| 312 | Male | 70 | G3 | T4a | N3a | M0 | ⅢC | 6×3.5×1.2 | 9 | Intestinal |
| 313 | Female | 56 | G2-G3 | T3 | N3a | M0 | ⅢB | 3×3×1 | 48 | Intestinal |
| 314 | Male | 59 | G2 | T4a | N2 | M0 | ⅢB | 5.5×3.5×2 | 14 | Intestinal |
| 315 | Male | 75 | G3 | T4b | N2 | M0 | ⅢC | 13×10×2.5 | 8 | Mixed |
| 316 | Male | 70 | G2 | T3 | N1 | M0 | ⅡB | 2.5×2×1.8 | 54 | Intestinal |
| 317 | Male | 71 | G2-G3 | T2 | N0 | M0 | ⅠB | 5.5×3×1 | 54 | Intestinal |
| 318 | Male | 63 | G3 | T3 | N2 | M0 | ⅢA | 7×6×1 | 17 | Diffuse |
| 319 | Male | 58 | G3 | T3 | N2 | M0 | ⅢA | 3×1.5×1 | 54 | Intestinal |
| 320 | Male | 75 | G3 | T3 | N2 | M0 | ⅢA | 6.5×6×3.5 | 7 | Intestinal |
| 321 | Male | 68 | G3 | T3 | N0 | M0 | ⅡA | 4×3×1.5 | 18 | Diffuse |
| 322 | Male | 57 | G2-G3 | T3 | N1 | M0 | ⅡB | 9×8×1.5 | 54 | Intestinal |
| 323 | Female | 47 | G3 | T3 | N2 | M0 | ⅢA | 5×4.5×1.5 | 15 | Diffuse |
| 324 | Female | 50 | G3 | T3 | N3b | M0 | ⅢB | 7.5×5×2.5 | 15 | Diffuse |
| 325 | Male | 47 | G3 | T3 | N3a | M0 | ⅢB | 10×6.5×1.8 | 15 | Diffuse |
| 326 | Male | 60 | G2-G3 | T4a | N3a | M0 | ⅢC | 6.5×6×1.5 | 53 | Intestinal |
| 327 | Male | 65 | G2-G3 | T2 | N0 | M0 | ⅠB | 6×4×2 | 53 | Intestinal |
| 328 | Male | 66 | G2-G3 | T4a | N0 | M0 | ⅡB | 4.5×4×1.5 | 53 | Intestinal |
| 329 | Male | 63 | G2 | T3 | N0 | M0 | ⅡA | 4×3×1.5 | 53 | Intestinal |
| 330 | Female | 76 | G3 | T3 | N1 | M0 | ⅡB | 6×4.5×2 | 24 | Intestinal |
| 331 | Male | 55 | G3 | T3 | N1 | M0 | ⅡB | 4.8×4×1 | 44 | Mixed |
| 332 | Female | 79 | G2-G3 | T4b | N2 | M0 | ⅢC | 12×11×2 | 53 | Intestinal |
| 333 | Female | 83 | G2-G3 | T4a | N2 | M0 | ⅢB | 4.5×4×1 | 30 | Mixed |
| 334 | Male | 60 | G3 | T3 | N2 | M0 | ⅢA | 1.5×1×0.5 | 49 | Intestinal |
| 335 | Female | 57 | G3 | T4a | N3b | M0 | ⅢC | 8×6×2 | 21 | Diffuse |
| 336 | Male | 54 | G3 | T3 | N3b | M0 | ⅢB | 6×3.5×1.2 | 9 | Intestinal |
| 337 | Male | 60 | G3 | T3 | N0 | M0 | ⅡA | 7×5×1.5 | 53 | Diffuse |
| 338 | Female | 52 | G2-G3 | T3 | N3a | M0 | ⅢB | 10×10×7 | 14 | Mixed |
| 339 | Male | 73 | G1-G3 | T3 | N3a | M0 | ⅢB | 10×8×6 | 34 | Diffuse |
| 340 | Female | 63 | G3 | T3 | N1 | M0 | ⅡB | 3×1.5×0.5 | 4 | Mixed |
| 341 | Female | 76 | G3 | T3 | N3a | M0 | ⅢB | 10×9×1.5 | 27 | Mixed |
